# Clinical Nurses' Job Embeddedness and Its Relationship to Professional Identity, Organizational Climate, and Compassion Fatigue: A Structural Equation Model

**DOI:** 10.1155/jonm/3700369

**Published:** 2025-09-08

**Authors:** Shuqi Zhai, Congcong Dai, Qinqin Liu, Yifan Lu, Chaoran Chen

**Affiliations:** ^1^Nursing Department of Huaihe Hospital, Henan University, Kaifeng, China; ^2^Institute of Nursing and Health, School of Nursing and Health, Henan University, Kaifeng, China

## Abstract

**Objective:** The purpose of this study was to examine the impact of professional identity on clinical nurses' job embeddedness and the role of organizational climate and compassion fatigue in this relationship.

**Background:** Nurses' job embeddedness has been a focus of attention in nursing, with lower levels of job embeddedness diminishing nurses' sense of commitment and belonging to their profession, increasing the risk of turnover, and affecting teamwork and continuity of care, thereby adversely affecting quality of care and patient safety. Therefore, improving the level of job embeddedness of nurses is important for stabilizing the nursing workforce and optimizing the quality of nursing services.

**Methods:** This study used a convenience sampling method to conduct a cross-sectional survey from July to October 2024 among 661 clinical nurses in three general hospitals in Henan Province. The survey instruments included the Nurse Professional Identity Scale, Organizational Climate Scale, Compassion Fatigue Scale, and Job Embeddedness Scale. The participants were certified, formally employed nurses capable of independently performing clinical duties and who voluntarily agreed to participate. Nurses who withdrew during the study, were unable to complete the survey due to special circumstances, or submitted incomplete questionnaires were excluded. Data analysis was conducted using SPSS 25.0, while AMOS 26.0 was used for model construction and mediation path analysis.

**Results:** The nurses' job embeddedness score was 21.93 ± 6.09, professional identity was a significant positive predictor of their job embeddedness (*r* = −0.437, *p* < 0.01), and organizational climate and compassion fatigue were chained mediators in their professional identity and job embeddedness (*β* = 0.034, *p* < 0.01). The mediator model showed a good fit.

**Conclusions:** Clinical nurses' professional identity is significantly positively correlated with their job embeddedness, and organizational climate and compassion fatigue play a chain mediating role between the two. Therefore, hospital administrators should pay attention to nurses' professional identity and take organizational climate and compassion fatigue as entry points to develop a series of targeted measures to enhance their job embeddedness.

## 1. Introduction

Job embeddedness refers to the closeness of the network of relationships formed between an individual and all job-related situations inside and outside the organization and is a key variable in predicting employee turnover behavior and career stability [1]. In the 1990s, Mitchell et al. first proposed job embeddedness theory [2], which suggests that job embeddedness consists of three main dimensions: linkage, fit, and sacrifice, which describe, respectively, the strength of an individual's relationships with co-workers, the organization, and the community; the match between values and organizational culture; and the potential losses that may be incurred by leaving the organization [3]. In recent years, researchers in the field of nursing have introduced job-embeddedness theory into nurse management, which suggests that a nurse's level of job-embeddedness is the sum of all the positive factors that maintain a nurse's retention in his or her position [4]. Increased job embeddedness not only helps to reduce nurses' willingness to leave [5] but also enhances nurses' sense of belonging and emotional commitment to the organization [6], thus optimizing the quality of nursing care and the patient's care experience [7]. In addition, studies have shown that job embeddedness not only directly influences nurses' levels of burnout but also mediates the relationship between professional self-concept and burnout, suggesting that enhancing job embeddedness can help alleviate nurse burnout [8]. Therefore, studying and improving nurses' job embeddedness has become an important issue in nursing management practice.

Currently, there are significant differences in the level of job embeddedness of nurses globally. A study in Egypt showed that most nurses scored high on job embeddedness [9]. Song's findings from a study in China also showed that clinical nurses reported moderate levels of job embeddedness, as evidenced by a low sense of organizational belonging and an increased willingness to leave their jobs [10]. Kim, on the other hand, found in her survey that Korean nurses have relatively low job embeddedness [11]. In comparison, studies in the United States show that about 89.6% of nurses report lower levels of job embeddedness [12]. Reasons for this phenomenon may include the overall pressure on the nursing profession, limited career development, mismatch of remuneration packages, and insufficient recognition of the nursing profession in the community [13]. Up to now, many national and international studies have explored the reasons affecting clinical nurses' job embeddedness, including organizational support [14], compensation packages [15], leadership styles [10], and work environments, as well as nurses' own personal job fit [16] and self-efficacy [17]. For example, studies have shown that good organizational support and development opportunities significantly increase nurses' level of job embeddedness [18], whereas excessive workload and lack of career advancement avenues weaken nurses' identification with the organization [19]. At the same time, the level of job embeddedness of nurses has a significant impact on both individuals and organizations. Higher levels of job embeddedness reduce turnover and enhance organizational commitment and career satisfaction [20], which improves the quality of care delivery and patient satisfaction [21]. Conversely, lower levels of job embeddedness may lead to higher nurse turnover [22], affecting the stability of the nursing team and negatively impacting patient safety and quality of care [23]. Therefore, an in-depth study of the job embeddedness of caregivers will not only help optimize the quality of care but also provide a scientific basis for healthcare policy development.

## 2. Background

### 2.1. Professional Identity and Job Embeddedness

Professional identity refers to an individual's cognitive and emotional identification with his or her profession, including the understanding and recognition of the values, goals, and meaning of the professional role. The concept was first introduced by Erikson in his theory of identity development [24]. Professional identity in the field of nursing is expressed as the degree of nurses' recognition of the intrinsic value of the nursing profession, its social significance, and its professional responsibility, and it is an important psychological motivation for nurses' professional behavior and career development [25]. Studies have shown that the level of professional identity has a significant impact on nurses' psychological state, job performance, and professional stability [26]. Nurses with higher levels of professional identity tend to show greater work engagement and higher career satisfaction, as well as being better able to adapt to the work environment and cope with professional challenges [27]. Additionally, professional identity has been found to directly impact nurses' job readiness and performance [28]. The relationship between professional identity and job embeddedness has also gained attention in nursing research. Evidence suggests that professional identity is a significant predictor of job embeddedness [29], as nurses with stronger identity are more aligned with organizational goals and experience a greater sense of connection and belonging [30]. Jin's [31] study further confirmed a significant positive correlation between professional identity and job embeddedness. However, although existing studies have explored the impact of professional identity on nurses' job embeddedness, there are still relatively few relevant studies and inconsistent findings. Based on this, we formulated Hypothesis 1.

**Hypothesis 1.**

There is a positive correlation between professional identity and job embeddedness.

### 2.2. The Mediating Role of Organizational Climate

Organizational climate refers to employees' overall perception and experience of the organization's internal environment, culture, and interpersonal relationships, including organizational support, teamwork, and communication atmosphere [32]. This concept, which emphasizes employees' psychological feelings and behavioral responses in the organizational environment [33], has received much attention and in-depth research in the healthcare field, especially in the nursing profession. Research has shown that the formation of organizational climate is a dynamic process, and its changes can directly affect nurses' job satisfaction, psychological state, and innovative behaviors [34]. As a dynamic and situational organizational characteristic, organizational climate plays a crucial role in shaping nurses' job satisfaction [35], physical and mental well-being [36], job performance [37], and willingness to leave [38]. It is a key factor in enhancing the quality of care and optimizing human resource management. Research has shown that nurses' professional identity has a significant impact on their organizational climate [39]. Feng's [40] study found a positive correlation between nurses' professional identity and organizational climate. Nurses with higher professional identity are more likely to engage in organizational activities and align with its culture, thereby fostering a positive organizational climate. A favorable climate, in turn, enhances nurses' identification with professional goals and strengthens their sense of fit and belonging [41]. Hashim [42] also confirmed a significant positive relationship between organizational climate and job embeddedness. Similarly, Lou [43] found that a more positive perceived organizational climate was associated with stronger job embeddedness among clinical nurses. These findings suggest that professional identity may indirectly influence job embeddedness by shaping organizational climate. Nurses with strong professional identities are more likely to integrate into the organizational culture and values, enhancing their sense of belonging and responsibility, which may further promote job embeddedness. Based on this, we formulated Hypothesis 2.

**Hypothesis 2.**

Organizational climate mediates the relationship between professional identity and job embeddedness.

### 2.3. The Mediating Role of Compassion Fatigue

The term compassion fatigue was first proposed by Joinson [44] in 1992 in the healthcare field to describe the phenomenon of exhaustion and dysfunction experienced by healthcare workers after experiencing prolonged periods of work-related stress [45]. Coetzee and Klopper [46] further noted that compassion fatigue is a gradual and cumulative process. As this state continues to intensify, it eventually leads to emotional exhaustion beyond the nurse's capacity [47]. Current compassion fatigue consists of two main dimensions: burnout and secondary traumatic stress. Studies have shown that as a negative emotional perception, compassion fatigue can have a range of negative impacts on nurses' physical and mental health [48, 49], the quality of clinical care [50], and the occurrence of presenteeism [51]. Studies have shown that professional identity is negatively associated with compassion fatigue in nurses [52]. Yi's [53] research on nursing interns also revealed a moderate negative correlation between professional identity and compassion fatigue, indicating that higher professional identity is linked to lower compassion fatigue. Similarly, Zhang et al. [54] found that occupational status serves as a protective factor, with stronger professional identity buffering the emotional toll of patient care. Compassion fatigue also significantly impacts nurses' job embeddedness. Research suggests a negative correlation between compassion fatigue and job embeddedness, with higher burnout associated with lower levels of embeddedness [55]. Nurses experiencing emotional stress from patient suffering often feel drained, lose motivation, and may develop turnover intentions, weakening their commitment and affecting care quality and career stability. Thus, compassion fatigue not only influences professional identity but is also a key predictor of job embeddedness. Based on this, we formulated Hypothesis 3.

**Hypothesis 3.**

Compassion fatigue mediates the relationship between professional identity and job embeddedness.

### 2.4. Relationship Between Organizational Climate and Compassion Fatigue

The Resource Theory of Work Demands [56] provides a solution based on the accumulation, protection, and replenishment of resources, which is of value in understanding the work behavior of employees and actively seeking positive protective factors. Organizational climate is considered an important environmental factor influencing employees' mental health and professional behavior [57]. Existing research suggests that nurses' organizational climate is significantly and positively correlated with their compassion fatigue and burnout [58]. When nurses feel the organization's recognition and support for their work, their psychological stress can be effectively relieved, and this supportive environment helps to protect and build up nurses' psychological resources [59]. Professional identity enhances nurses' perception of organizational climate by reinforcing their sense of self-worth and belonging. Nurses with strong professional identities are more likely to feel integrated and supported within the team, reducing stress and the risk of compassion fatigue. In contrast, prolonged compassion fatigue depletes psychological resources, weakens resilience, and increases vulnerability to work-related stress, ultimately reducing job embeddedness. Based on the above literature review, we proposed Hypothesis 4.

**Hypothesis 4.**

Organizational climate and compassion fatigue play a chain mediating role between professional identity and job embeddedness.

**Figure 1:**
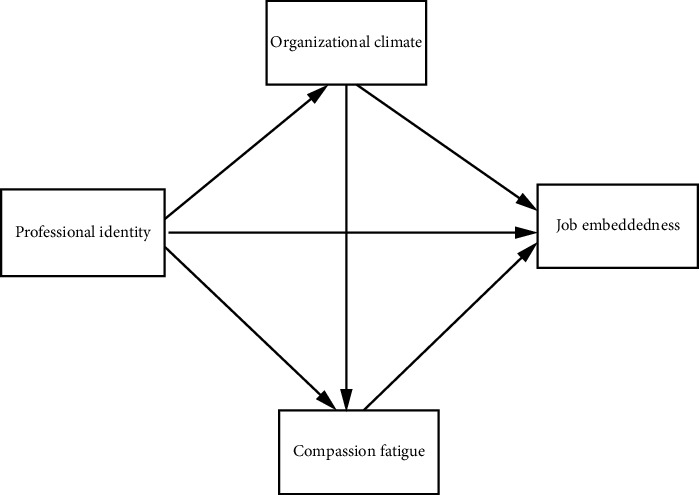
Conceptual framework and hypothesis.

The purpose of this study was to explore the relationship between professional identity and clinical nurses' job embeddedness and the mediating role of organizational climate and compassion fatigue in this relationship. Based on the above discussion, the conceptual framework constructed for this study is shown in Figure 1.

## 3. Methods

### 3.1. Study Design

This study conducted a cross-sectional survey of clinical nurses in three hospitals in Henan Province, China, from July 2024 to October 2024 using a convenience sampling method to examine the relationship between clinical nurses' professional identity, organizational climate, compassion fatigue, and job embeddedness.

### 3.2. Participants

In this study, clinical nurses from three hospitals in Henan Province were surveyed using a convenience sampling method. Clinical nurses voluntarily participated in the study after fully understanding the purpose and significance of the study and obtaining informed consent from their leaders. The inclusion criteria for the study subjects were as follows: (1) certified and registered nurses; (2) formally employed and able to perform clinical duties independently; and (3) voluntarily participated in this study based on informed consent. The exclusion criteria were as follows: (1) nurses who withdrew from the study in the middle of the study; (2) nurses who were unable to participate due to special circumstances that occurred during the survey; and (3) questionnaires with incomplete responses.

### 3.3. Data Collection

Data were collected via an online questionnaire survey conducted from July 15 to August 1, 2024. Prior to data collection, the research team contacted the directors of nursing departments at the participating hospitals to explain the purpose, target population, and procedures of the study. After obtaining approval, an electronic questionnaire link was distributed to head nurses of each unit, who then invited eligible nurses to participate based on the inclusion criteria. The first page of the questionnaire included a standardized introduction that explained the study's purpose and instructions for completion and emphasized the voluntary and anonymous nature of participation, allowing respondents to withdraw at any point. To ensure data completeness and uniqueness, all items were set as mandatory, and each account or device could submit the questionnaire only once. The research team monitored the submission process in real time and performed data screening upon collection. Questionnaires completed in less than 100 s or showing highly uniform responses were excluded as invalid. A total of 700 questionnaires were distributed, and 661 valid responses were retained after excluding 39 invalid submissions, yielding a valid response rate of 94.4%.

### 3.4. Measurement

#### 3.4.1. Demographic Characteristics

Demographic characteristics collected included gender, age, marital status, education level, type of hospital, department of work, job title, mode of employment, length of service, monthly income, hours of work per day, and average number of night shifts per month.

#### 3.4.2. Professional Identity

This study used the Nurses' Professional Identity Rating Scale developed by Liu et al. [60] to measure the professional identity of new nurses. The scale consists of 30 items that test five dimensions: assessment of occupational perceptions (9 items), occupational social support (6 items), occupational social skills (6 items), occupational frustration coping (6 items), and occupational self-reflection (3 items). It uses a Likert-5 scale ranging from 1 (*very non-compliant*) to 5 (*very compliant*). The scale is scored out of 150, with higher scores indicating a higher level of professional status for nurses. The Cronbach's α coefficient for this scale is 0.938 [60]. In this study, the Cronbach's α coefficient was 0.982.

#### 3.4.3. Organizational Climate

This study used the Nursing Organization Classification Scale developed by He Liping and Jing [61]. The scale consists of 24 items in four dimensions: equitable supportive behaviors (10 items), coworker behaviors (5 items), interpersonal climate behaviors (4 items), and intimate and aggressive climate behaviors (5 items). The scale is rated on a 5-point Likert scale ranging from 1 to 5, with scores ranging from “*very inconsistent*” to “*very consistent*.” The scale has a total score of 24–120, with higher scores indicating a better organizational climate. The Cronbach's α coefficient for this scale is 0.927 [61]. In this study, the Cronbach's α coefficient was 0.971.

#### 3.4.4. Compassion Fatigue

This study used the Compassion Fatigue Short Scale (CF-Short Scale) to assess the compassion fatigue levels of clinical nurses. The scale was developed by Adams et al. [62] and translated by Paula Lou. The scale consists of 2 dimensions, secondary trauma and burnout, with a total of 13 entries. Secondary trauma consists of 5 entries, and burnout consists of 8 entries. The scale uses a 10-point Likert scoring system, with a maximum score of 130. A higher total score indicates a more severe level of compassion fatigue. The level of compassion fatigue can be classified into three levels based on the mean score of the entries: *mild* (< 4 points), *moderate* (4–7 points), and *severe* (> 7 points), and the validated factor analysis showed that the questionnaire had good construct validity, and the Cronbach's α coefficient of the scale was 0.90 [62]. In this study, the Cronbach's α coefficient was 0.970.

#### 3.4.5. Job Embedding

This study used the Job Embeddedness Scale developed by Crossley [63] and translated and Chineseized by Mei [64]. The scale is unidimensional and consists of 7 items. The scale was rated on a 5-point Likert scale ranging from “1” to “5,” indicating *not at all* to *perfect*. Entries 4 and 6 are reverse scored. The scale was rated on a 7–35 scale, with higher scores indicating greater job embeddedness of the nurses. The Cronbach's α coefficient for this scale is 0.890 [64]. In this study, the Cronbach's α coefficient was 0.864.

### 3.5. Ethical Considerations

First, this study is a cross-sectional study without clinical experiments and has no impact on the body and mind of the participants. Second, before filling out the questionnaire, we informed the participants about the purpose and importance of the study, and they were able to drop out at any time. Third, we will not disclose information about the participants, and the data and information collected were only used for this study. The study obtained the informed consent of all study participants. Finally, this research was supported by the relevant ethics committee (ID no.: HUSOM2023-478).

### 3.6. Statistical Analysis

In this study, SPSS 25.0 and AMOS 26.0 were utilized for data analysis and model construction. First, descriptive statistics were used in this study to assess the demographic characteristics of the participants, as well as their levels of professional identity, organizational climate, compassion fatigue, and job embeddedness. Second, tests indicated that the data collected in this study followed a normal distribution, and the variables exhibited linear correlations. Therefore, Pearson correlation analysis was used to examine the relationships among the four variables. In addition, AMOS 26.0 was used to map the model and explore the relationships and parameters among the variables. This study conducted chi-square tests and assessed the model fit using the chi-square/degree of freedom (*χ*^2^/df), Comparative Fit Index (CFI), Adjusted Goodness of Fit Index (AGFI), Root Mean Square Error of Approximation (RMSEA), Goodness of Fit Index (GFI), Incremental Fit Index (IFI), and Tucker–Lewis Index (TLI) to evaluate the overall fit of the hypothesized model. The smaller the *χ*^2^/df value, the better the model fit. A smaller RMSEA indicates a better-fitting model. For GFI, TLI, CFI, and AGFI, values range from 0 to 1, with values closer to 1 indicating a better fit [65]. Finally, this study calculated the 95% confidence interval (CI) for the bias-corrected percentile bootstrap from 5000 bootstrap samples [66]. The *p* value was two-tailed, and values below 0.05 were considered statistically significant.

## 4. Results

### 4.1. The Demographic Characteristics of the Participants

A total of 661 nurses responded to the survey. Characteristics of the study participants are summarized in Table 1. Among the 661 participants, 85.2% were female and 14.8% were male; 43.1% were between the ages of 26–35 and 34.3% were between the ages of 36–46; 70.8% were married; 76.1% had a bachelor's degree; 83.2% worked in a tertiary care hospital; 35.4% had the professional title of senior nurse; 35.1% were in internal medicine; 54.8% worked under contract; 57.6% had an average monthly income of RMB 4001–7000; 66.7% worked 8–10 h a day; and 40.8% of the participants worked an average of 5–8 night shifts per month.

### 4.2. Pearson's Correlation Analysis

The results of the mean, standard deviation, and correlation coefficient for each variable in this study are shown in Table 2. The scores for professional identity, organizational climate, compassion fatigue, and job embeddedness were 92.11 ± 28.48, 76.35 ± 20.67, 67.35 ± 25.99, and 21.93 ± 6.09, respectively. In addition, Pearson's correlation analysis found a positive correlation between professional identity and organizational climate (*r* = 0.422, *p* < 0.01) and a negative correlation with compassion fatigue (*r* = −0.676, *p* < 0.01) and job embeddedness (*r* = −0.437, *p* < 0.01). There was a negative correlation between organizational climate and compassion fatigue (*r* = −0.552, *p* < 0.01) and a positive correlation with job embeddedness (*r* = 0.397, *p* < 0.01). In addition, there was a significant negative correlation between compassion fatigue and job embeddedness (*r* = −0.459, *p* < 0.01).

### 4.3. Mediating Effect Analysis and Hypothesis Path Testing

**Figure 2:**
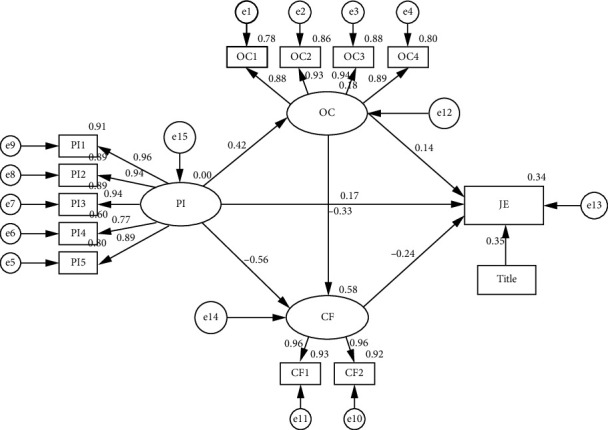
Path analysis diagram of professional identity, organizational climate, compassion fatigue, and job embeddedness. Note: PI, professional identity; PI1, evaluation of career perceptions; PI2, professional social skills; PI3, professional social support; PI4, career frustration response; PI5, professional self-reflection. OC, organizational climate; OC1, support for fair behavior; OC2, colleague behavior; OC3, interpersonal climate behavior; OC4, intimate and aggressive climate behavior. CF, compassion fatigue; CF1, professional boredom; CF2, secondary traumatic stress. JE, job embeddedness.

In this study, AMOS was mainly used to analyze intermediary paths [67], and the results are shown in Figure 2 and Table 3. As shown in Figure 2, professional identity was a significant positive predictor of nurses' organizational climate and job embeddedness (*β* = 0.42, *p* < 0.001; *β* = 0.17, *p* < 0.001) and a significant negative predictor of their compassion fatigue (*β* = −0.56, *p* < 0.001). Organizational climate positively predicted job embeddedness (*β* = 0.14, *p* < 0.001), while organizational climate was a significant negative predictor of compassion fatigue (*β* = −0.33, *p* < 0.001). Compassion fatigue negatively predicted job embeddedness (*β* = −0.24, *p* < 0.001).

Subsequently, we made bootstrap samples as well as bias-corrected CIs to examine the mediating effects of organizational climate and compassion fatigue on the relationship between nurses' professional identity and job embeddedness. The 95% CI for percentile bootstrapping and bias-corrected percentile bootstrapping were calculated from 5000 bootstrap samples. The results indicated that organizational climate and compassion fatigue significantly mediated this relationship, with a fully mediated effect of 0.226. Specifically, the fully mediated effect consisted of three indirect pathways.

The first path was the indirect effect of professional identity on nurses' job embeddedness through organizational climate, that is, professional identity ⟶ organizational climate ⟶ job embeddedness. The indirect effect was 0.059, bootstrap 95% CI: (0.027, 0.100), *p* < 0.001.

The second path was the indirect effect of professional identity on nurses' job embeddedness through compassion fatigue, that is, professional identity ⟶ compassion fatigue ⟶ job embeddedness. The indirect effect was 0.134, bootstrap 95% CI: (0.068, 0.205), *p* < 0.001.

The third path was a chain-mediated effect of organizational climate and compassion fatigue, with the path being professional identity ⟶ organizational climate ⟶ compassion fatigue ⟶ job embeddedness. The effect value was 0.034, bootstrap 95% CI: (0.017, 0.055), *p* < 0.001.

### 4.4. Model Fit Analysis

Table 4 shows the resulting modeling of the structural equations. This study used AMOS 26.0 to test for mediating effects, and after including professional identity, organizational climate, compassion fatigue, and job embeddedness in the structural equation modeling analysis, the model fit indices were *χ*^2^/df = 3.144 (< 5.0), GFI = 0.959 (> 0.90), CFI = 0.986 (> 0.90), AGFI = 0.938 (> 0.90), TLI = 0.981 (> 0.90), IFI = 0.986 (> 0.90), and RMSEA = 0.057 (< 0.08), indicating a good model fit.

## 5. Discussion

This study explored the relationship between professional identity, organizational climate, compassion fatigue, and job embeddedness. The results showed that clinical nurses had a moderately high job embeddedness score (21.93 ± 6.09), consistent with previous studies [15, 68], suggesting that this group has a sense of belonging and a willingness to sustain commitment in their positions. A significant positive correlation was found between professional identity and job embeddedness, indicating that nurses with higher levels of professional identity tend to exhibit stronger job embeddedness, which aligns with the findings of Lu et al. [30]. When nurses have a high level of identification with the nursing profession, their intrinsic work motivation and psychological resources are fully stimulated, leading to a more positive integration into the work environment and a solid sense of work embeddedness [31]. However, when nurses are confronted with prolonged high-pressure work environments and professional challenges, their professional identity may be affected to some extent, thus reducing the level of job embeddedness [69]. Excessively low professional identity not only weakens nurses' sense of attachment to their work environment but may also trigger burnout and a tendency to leave, which in turn negatively affects the overall quality of care in hospitals [70]. Therefore, enhancing nurses' professional identity and job embeddedness should be a key focus of hospital management. First, hospitals should support career development by providing clear promotion pathways, job rotation systems, and opportunities for professional growth to encourage long-term commitment. Second, optimizing the work environment—through reasonable shift scheduling, manageable workloads, and competitive compensation—can improve job satisfaction and stability. Third, nurses should make full use of training opportunities to update their knowledge and skills, build confidence, and actively engage in teamwork to strengthen communication and a sense of professional responsibility. Through joint efforts from both hospitals and nurses, professional identity and job embeddedness can be effectively improved, promoting the sustainable development of the nursing profession.

This study found that organizational climate was a mediating variable for clinical nurses' professional identity and job embeddedness, and Hypothesis 2 was tested. There is a significant positive correlation between professional identity and organizational climate, and nurses with higher levels of professional identity usually create a good organizational climate, which is consistent with Feng's findings [40]. Nurses who highly identify with their profession internalize nursing values and standards, leading to greater responsibility and dedication, which contributes to a positive organizational culture [71]. Furthermore, high professional identity strengthens organizational commitment, promoting teamwork, communication, and collaboration, thus creating a supportive work atmosphere [72]. In addition, this study found that a good organizational climate not only enhances nurses' professional identity with their professional goals but also increases their sense of fit and belonging to the organization [41]. Clinical nurses who perceived a more positive climate reported higher levels of job embeddedness, which aligns with previous research [43]. Hashim [42] also emphasized the positive link between organizational climate and job embeddedness. A supportive team environment fosters emotional attachment and a sense of irreplaceability, enhancing the affective dimension of job embeddedness [73]. These emotional bonds further strengthen intrinsic motivation and improve nurses' work engagement and efficiency [74]. These findings suggest that improving clinical nurses' perception of organizational climate requires efforts at the individual, team, and management levels. At the individual level, nurses should actively embrace teamwork, communicate openly, and align with the organizational culture. At the team level, team building should be strengthened by encouraging mutual support and organizing regular case discussions, experience sharing, and group activities to foster a positive and cohesive work environment. At the management level, hospitals should implement fair performance evaluations and incentive mechanisms to ensure nurses' efforts are recognized. Additionally, open communication channels should be maintained to address nurses' concerns, adjust strategies promptly, and enhance their sense of trust and belonging. These combined efforts can create a supportive organizational climate, improve job satisfaction and teamwork, and ultimately enhance the overall quality of nursing care.

This study found that compassion fatigue was a mediating variable for clinical nurses' professional identity and job embeddedness, and Hypothesis 3 was tested. A significant negative correlation was found between professional identity and compassion fatigue, with nurses who exhibit stronger professional identity reporting lower levels of compassion fatigue, consistent with previous research [75, 76]. As noted by Geoffrion [77], prolonged exposure to trauma can alter nurses' worldviews and weaken their self-perception of the profession, negatively affecting attitudes, values, and mental health. In contrast, nurses with high professional identity tend to internalize the value and meaning of their work, which provides emotional support and motivation when facing patient suffering [76]. This internalization enhances psychological resilience and promotes positive coping strategies, helping to reduce emotional exhaustion [54]. Conversely, nurses with low professional identity may lack intrinsic motivation and effective support, making them more vulnerable to compassion fatigue. Furthermore, the study found a significant negative correlation between compassion fatigue and job embeddedness—nurses experiencing higher compassion fatigue typically showed lower job embeddedness—aligning with previous findings [13, 55]. Nursing staff's attachment and sense of belonging to the work environment decrease significantly after prolonged emotional exhaustion, which in turn weakens job embeddedness and affects teamwork and information sharing [78]. Nurses with lower compassion fatigue were able to remain emotionally stable and cope positively when faced with patients' emotional stress, which made them more likely to receive organizational support and mutual assistance from colleagues. This psychological adjustment mechanism helps to increase nurses' work engagement and willingness to stay in the workforce, further enhancing the overall level of work embeddedness [79]. Conversely, nurses with high levels of compassion fatigue tend to have difficulty establishing deep attachment and identification with the organization due to the depletion of emotional resources and increased psychological burden, leading to decreased job embeddedness. Based on the research findings, enhancing nurses' compassion satisfaction and job embeddedness requires coordinated interventions at multiple levels. At the hospital level, administrators should optimize the work environment, foster a culture of respect and support, improve facilities and equipment, allocate nursing staff appropriately, and regularly conduct mental health training to strengthen psychological support for nurses. At the departmental level, head nurses should focus on creating a positive and collaborative team atmosphere, implement flexible scheduling to balance workload and rest, and regularly organize intra-departmental communication and cooperation activities to promote mutual understanding among team members, thereby further enhancing nurses' sense of professional belonging and team cohesion. Individually, nurses are encouraged to practice self-care, engage in emotional regulation and skills training, and build peer support networks to alleviate compassion fatigue and enhance job embeddedness.

This study also found that the influence of professional identity on clinical nurses' job embeddedness could be mediated through the chain mediating effects of organizational climate and compassion fatigue, which was expected to be consistent with Hypothesis 4. When nurses have a high level of professional identity, they are more likely to internalize nursing values and professional standards and actively participate in organizational activities, thus creating a positive and harmonious organizational climate [72]. A good organizational climate not only enhances nurses' sense of belonging and organizational commitment but also effectively alleviates compassion fatigue and keeps them in a good psychological state when facing emotional challenges [74]. In turn, the reduction of compassion fatigue helps nurses to be more focused and consistently engaged in their work, enhancing their sense of attachment to their position and job embeddedness [55]. On the contrary, nurses with lower professional identity tend to have difficulty in creating a good organizational climate and are prone to accumulate compassion fatigue, which ultimately weakens their level of job embeddedness. Therefore, enhancing nurses' professional identity can both optimize organizational climate and reduce compassion fatigue, thereby effectively enhancing job embeddedness and promoting nursing quality.

## 6. Limitations and Future Research

There are some limitations of this study. First, the subjects of this study were all nurses in Henan Province, and the sample could not represent all clinical nurses in different regions, which led to the generalization of the results; in the future, multicenter studies can be conducted on hospitals in different regions to promote the generalization of the results. Secondly, this study used self-administered questionnaires to collect data, and the results were subjective; future research could use a combination of qualitative interviews and other-assessment questionnaires to collect data in a variety of ways. Finally, this study was a cross-sectional investigation and could not verify the dynamic development and causality that occurred between the variables, and the mechanism of action between these variables could be explored in further detail in the future using a longitudinal study approach. Finally, since job embeddedness is a multidimensional issue and can be influenced by various factors, it is recommended that future research also consider other potential contributors to burnout, such as work shift systems, workload, sleep disorders, and psychological conditions.

## 7. Conclusion

This study builds a theoretical framework for research from the perspective of clinical nurses and explores the processes and mechanisms by which professional identity influences job embeddedness in clinical nurses. It was found that there was a significant positive effect of professional identity on nurses' job embeddedness, with organizational climate and compassion fatigue acting as chain mediators between professional identity and nurses' job embeddedness. Therefore, hospital administrators should take targeted interventions to continuously optimize the organizational climate of the nursing team, reduce the phenomenon of compassion fatigue, and enhance nurses' sense of professional identity and job embeddedness so as to improve the stability of the nursing team and the quality of nursing services.

## Figures and Tables

**Table 1:** Demographic characteristics of the participants (*N* = 661).

Variables		*N*	%
Gender	Male	98	14.8
	Female	563	85.2

Educational background	Junior college	86	13.0
	Bachelor's	503	76.1
	Master's or above	72	10.9

Years	≤ 25	96	14.5
	26–35	285	43.1
	36–45	227	34.3
	≥ 46	53	8.1

Marital status	Single	168	25.4
	Married	468	70.8
	Divorced or widowed	25	3.8

Hospital level	Tertiary	550	83.2
	Secondary or below	111	16.8

Professional title	Nurse	170	25.7
	Senior nurse	234	35.4
	Nurse-in-charge	216	32.7
	Deputy chief nurse or above	41	6.2

Departments	Internal medicine	232	35.1
	Surgery	172	26.1
	Obstetrics and gynecology	49	7.4
	Pediatrics	34	5.1
	Emergency department	54	8.2
	Outpatient department	47	7.1
	Intensive care unit	32	4.8
	Operative department	41	6.2

Labor and personnel relations	Formal establishment	116	17.5
	Personnel agency	147	22.2
	Contract system	363	54.9
	Labor dispatch	35	5.3

Years of working (years)	< 2	98	14.8
	2–5	144	21.8
	6–10	108	16.3
	> 10	311	47.1

Monthly income (RMB)	< 4000	122	18.5
	4001–7000	381	57.6
	7001–10000	131	19.8
	> 10,000	27	4.1

Daily working hours (h)	< 8	115	17.4
	8–10	441	66.7
	> 10	105	15.9

Average number of night shifts per month (times)	0	105	15.9
	1–4	217	32.8
	5–8	270	40.8
	> 8	69	10.5

**Table 2:** Descriptive statistics and correlation analysis (*N* = 661).

	PI	OC	CF	JE	Mean	Standard deviation
PI	1				92.11	28.48
OC	0.422^∗∗^	1			76.35	20.67
CF	−0.676^∗∗^	−0.552^∗∗^	1		67.35	25.99
JE	−0.437^∗∗^	0.397^∗∗^	−0.459^∗∗^	1	21.93	6.09

Abbreviations: CF, compassion fatigue; JE, job embeddedness; OC, organizational climate; PI, professional identity.

^∗∗^ *p* < 0.01 (two-tailed).

**Table 3:** Total, direct, and indirect effects of professional identity on job embeddedness (*N* = 661).

Effect	Path relationship	Effect	Bootstrap SE	Bootstrap 95% CI	*p* value
Direction effect	PI ⟶ JE	0.174	0.051	0.075, 0.273	0.001

Indirection effect	PI ⟶ OC ⟶JE	0.059	0.018	0.027, 0.100	0.001
	PI ⟶ CF ⟶ JE	0.134	0.035	0.068, 0.205	0.001
	PI ⟶ OC ⟶ CF ⟶ JE	0.034	0.009	0.017, 0.055	0.001

Total mediating effect		0.226	0.040	0.149, 0.307	0.001

Total effect	PI ⟶ JE	0.400	0.032	0.336, 0.462	0.001

Abbreviations: CF, compassion fatigue; JE, job embeddedness; OC, organizational climate; PI, professional identity.

**Table 4:** Model fit summary.

Model fit	*χ* ^2^/df	GFI	AGFI	RMR	NFI	RFI	IFI	TLI	CFI	RMSEA
	3.144	0.959	0.938	0.293	0.979	0.973	0.986	0.981	0.986	0.057

## Data Availability

The data that support the findings of the study are available from the corresponding author upon reasonable request.
